# Intracranial pressure responsiveness to positive end-expiratory pressure in different respiratory mechanics: a preliminary experimental study in pigs

**DOI:** 10.1186/s12883-018-1191-4

**Published:** 2018-11-05

**Authors:** Han Chen, Jing Zhou, Yi-Qin Lin, Jian-Xin Zhou, Rong-Guo Yu

**Affiliations:** 1 Surgical Intensive Care Unit, Fujian Provincial Clinical College, Fujian Medical University, No 134, Dongjie Street, Gulou District, Fuzhou, 350001 Fujian China; 2 Department of Critical Care Medicine, Beijing Tiantan Hospital, Capital Medical University, Beijing, China

**Keywords:** Intracranial pressure, Respiratory mechanics, Lung injury, Chest wall, Elastance, Esophageal pressure, Positive end-expiratory pressure

## Abstract

**Background:**

Respiratory mechanics affects the effect of positive end-expiratory pressure (PEEP) on intracranial pressure (ICP). Respiratory mechanics of the lung and the chest wall was not differentiated in previous studies. In the present study, we investigated the influence of the following possible determinants of ICP responsiveness to PEEP: chest wall elastance (E_CW_), lung elastance (E_L_), and baseline ICP.

**Methods:**

Eight healthy Bama miniature pigs were studied. The increase of E_L_ was induced by instillation of hydrochloride, and the increase of E_CW_ was induced by strapping the animals’ chest wall and abdomen. A balloon-tipped catheter was placed intracranially for inducing intracranial hypertension. Six experimental conditions were investigated in sequence: 1) *Normal*; 2) *Stiff Chest Wall*; 3) *Lung Injury*; 4) *Lung Injury* + *Stiff Chest Wall*; 5) *Lung Injury* + *Stiff Chest Wall + Intracranial Hypertension* and 6) *Lung Injury* + *Intracranial Hypertension*. PEEP was gradually increased in a 5 cm H_2_O interval from 5 to 25 cm H_2_O in each condition. Blood pressure, central venous pressure, ICP, airway pressure and esophageal pressure were measured.

**Results:**

Hydrochloride instillation significantly increased E_L_ in conditions with lung injury. E_CW_ significantly increased in the conditions with chest wall and abdomen strapping (all *p* <  0.05). ICP significantly increased with increments of PEEP in all non-intracranial hypertension conditions (*p* <  0.001). The greatest cumulative increase in ICP was observed in the *Stiff Chest Wall* condition (6 [5.3, 6.8] mm Hg), while the lowest cumulative increase in ICP was observed in the *Lung Injury* condition (2 [1.3, 3.8] mm Hg). ICP significantly decreased when PEEP was increased in the intracranial hypertension conditions (*p* <  0.001). There was no significant difference in cumulative ICP change between the two intracranial hypertension conditions (*p* = 0.924).

**Conclusions:**

Different respiratory mechanics models can be established via hydrochloride induced lung injury and chest wall and abdominal strapping. The effect of PEEP on ICP is determined by respiratory mechanics in pigs with normal ICP. However, the responsiveness of ICP to PEEP is independent of respiratory mechanics when there is intracranial hypertension.

## Background

It has been reported that a significant portion of brain-injured patients can develop pulmonary complications including acute respiratory distress syndrome (ARDS) and neurogenic pulmonary edema [1–5]. Mechanical ventilation is needed in this population, and positive end-expiratory pressure (PEEP) is used to improve oxygenation as well as to recruit and/or prevent alveolar collapse [6–8].

However, there have long been concerns that the use of PEEP in brain-injured patients could cause increase of intracranial pressure (ICP) and deteriorate neurological status, especially in those who are with signs of cerebral edema. Previous studies yielded conflicting results in the effects of PEEP on ICP, with ICP increasing [9–13], not markedly changing [14–16] or even decreasing [17] after the application of PEEP, suggesting a multifactorial mechanism. Several possible determinants for the effect of PEEP on ICP have been proposed, including baseline ICP [11], intracranial compliance [12, 13] and respiratory mechanics [9, 10].

Theoretically, PEEP can increase ICP via elevating intrathoracic pressure and diminishing venous return [18, 19], where the transmission of PEEP into the thoracic cavity depends on the respiratory mechanics. Chapin and colleges reported that increased lung elastance (E_L_) and decreased chest wall elastance (E_CW_) can minimize the effect of PEEP on pleural pressure [20]. Clinical studies have also suggested that the effect of PEEP on ICP is attenuated when respiratory system elastance (E_RS_) increases. However, E_L_ and E_CW_ were not differentiated in these studies [9, 10]. For a given increased E_RS_, it might be attributed to either the increase in E_L_ due to pulmonary disease (e.g. ARDS), or the increase in E_CW_ due to chest wall impairment (e.g. intra-abdominal hypertension or massive pleural effusion), or both. It has been shown that the E_CW_ to E_RS_ ratio (E_CW_/E_RS_ ratio) varied from 0.2 to 0.8 in mechanically ventilated patients [21]. Therefore, it is important to clarify the mechanical characteristics of both the lung and the chest wall when investigating the effects of PEEP on ICP.

Although it is known that the decrease of E_CW_/E_RS_ ratio can attenuate the effect of PEEP to pleural pressure [20], it is still unknown whether it can attenuate the effect of PEEP to ICP. We hypothesized that a greater E_CW_/E_RS_ ratio would result in a greater ICP responsiveness to increased PEEP. In this preliminary study, we investigated the influence of the following possible determinants of ICP responsiveness to PEEP: the elevated E_CW_, which increases E_CW_/E_RS_ ratio; the elevated E_L_, which reduces E_CW_/E_RS_ ratio; and the elevated baseline ICP.

## Methods

### Animal preparation

Eight healthy, male Bama miniature pigs (weight 10–20 kg, mean 13.6 kg) were studied. All animals received humane care in compliance with the National Institutes of Health guidelines for the care and use of experimental animals and with the approval of the Institutional Review Board of Fujian Provincial Hospital (Approval # KY − 2016010). Animals were purchased from Guangxi University. Animals were fasted preoperatively. Premedication consisted of intramuscular 10 − 20 mg/kg ketamine, followed by an intravenous bolus of 10 mg midazolam. Continuous sedation consisted of intravenous 0.2–1.0 mg/kg/hr midazolam and 0.1–0.2 mcg/kg/hr fentanyl. Maintenance fluid was administrated (lactated Ringer’s solution, 5 mL/kg/hr); additional fluids and catecholamine infusions were not allowed. Animals were placed on a heating pad in supine position. Central venous catheter was placed via right internal jugular vein for fluid infusion and central venous pressure (CVP) measurements. Arterial cannula was placed via right femoral artery to measure blood pressure. Tracheotomy was performed and a 5.5–6.5 tracheotomy cuffed tube was placed. Animals were than paralyzed via intravenous infusion of 10 mg vecuronium bromide and mechanically ventilated with a Servo-s ventilator (Maquet, Solna, Sweden). Vecuronium bromide was continuously infused (1 mg/kg/hr) and an additional 5 mg bolus was administrated if there was spontaneous breathing effort, which was determined by a negative deflection in the esophageal pressure (P_ES_) tracing.

Animals were than turned to right lateral position. A midline, transverse incision was performed along the dorsal surface of the head to expose the underlying skull. One burr hole was created approximately 10 mm left/lateral of midline and 10 mm anterior to the coronal suture. An intraparenchymal ICP monitor catheter (Codman Microsensor, Raynham, MA, USA) was placed through this opening. The distal tip of the catheter was placed in the exposed cortex at a depth of 1 cm. A bedside ICP monitor (Codman ICP Express, Raynham, MA, USA) was connected. Another burr hole was created approximately 10 mm right/lateral of midline and 10 mm anterior to the coronal suture. A balloon-tipped catheter (5 mL, 8Fr Foley) was placed through the hole for inducing intracranial hypertension (IH).

Animals were turned back to supine position. A SmartCath-G esophageal balloon catheter (7003300, CareFusion Co., Yorba Linda, CA, USA) was placed to measure P_ES_. A positive pressure occlusion test was used to confirm the proper balloon position [22, 23]. P_ES_ and airway pressure (P_AW_) were measured by two KT 100D-2 pressure transducers (KleisTEK di CosimoMicelli, Italy, range: +/− 100 cm H_2_O).

### Experimental protocol

**Fig. 1:**
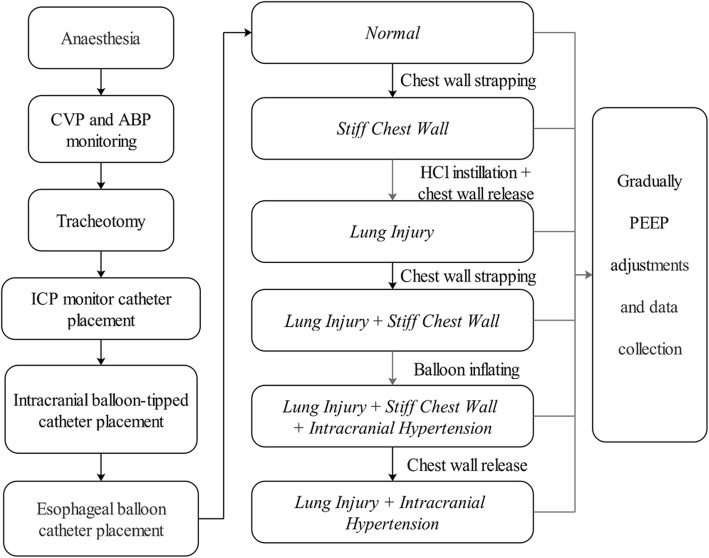
Experimental flowchart

Six experimental conditions were investigated in sequence (Fig. 1):

1. *Normal* condition;
2. *Stiff Chest Wall* experimental condition (*CW* condition);
3. *Lung Injury* experimental condition (*L* condition);
4. *Lung Injury* + *Stiff Chest Wall* experimental condition (*L + CW* condition);
5. *Lung Injury* + *Stiff Chest Wall + IH* experimental condition (*L + CW + IH* condition);
6. *Lung Injury* + *IH* experimental condition (*L + IH* condition).

In each condition, PEEP was gradually increased in a 5 cm H_2_O interval from 5 to 25 cm H_2_O. Animal was ventilated for five minutes after each PEEP increment to allow ICP stabilization. Measurements were taken after the five-minute stabilization (see below). After the last measurement of one condition (25 cmH_2_O of PEEP), PEEP was decreased to 5 cmH_2_O and the preparation of the next condition was completed under a PEEP setting of 5 cmH_2_O. All ventilator settings except PEEP were not changed during the entire experiment. The pig was euthanized in the end of the experiment by overdose pentobarbital injection (100 mg/kg).

The increase of E_CW_ was induced by strapping the animals’ chest wall and abdomen with an inelastic, adjustable bellyband. In addition, two pneumatic cuffs were placed between the bellyband and the abdomen as well as the chest wall [24, 25]. The bellyband was adjusted so that inspirations were not hampered when the pneumatic cuffs were not inflated (normal E_CW_). To increase E_CW_, the pneumatic cuffs were inflated to a pressure of 20 cm H_2_O [24, 25].

The increase of E_L_ was induced by slowly instillation of 0.1 mol/L hydrochloride (4 mL/kg) down the endotracheal tube via a thin suction catheter placed at the level of the carina. This method has been previously reported as creating an animal model with ARDS-like lung injury including lung inflammation, edema, hemorrhage, and variable lung region aeration [25, 26]. One hour following hydrochloride administration the model was validated by achieving a pulse oxygen saturation ≤ 90%.

IH was induced by inflating the intracranial balloon with saline at a rate of 0.5 mL/min until the ICP was constant between 30 and 40 cm H_2_O for > 30 min [26, 27].

### Measurements

Mechanical ventilation was set as volume-controlled ventilation with a constant flow, an inspiratory to expiratory ratio of 1:2, a tidal volume (V_T_) of 10 mL/kg, a respiratory rate of 20 breaths/min and an inspired oxygen fraction of 100%. ICP, mean arterial pressure (MAP), cerebral perfusion pressure (CPP, calculated as MAP minus ICP) and CVP were measured. At each tested PEEP level, end-inspiratory and end-expiratory occlusion were performed, each for 3 s. P_ES_ and P_AW_ during the last second of occlusion were recorded. Respiratory mechanics were calculated as follows:

$$ {\mathrm{E}}_{\mathrm{RS}}=\frac{{\mathrm{P}}_{\mathrm{P}\mathrm{LAT}}-{\mathrm{P}\mathrm{EEP}}_{\mathrm{T}\mathrm{OTAL}}}{{\mathrm{V}}_{\mathrm{T}}} $$

Where P_PLAT_ and PEEP_TOTAL_ represent P_AW_ at end-inspiratory and end-expiratory occlusion, respectively.

$$ {\mathrm{E}}_{\mathrm{CW}}=\frac{{\mathrm{P}}_{\mathrm{E}\mathrm{S}-\mathrm{EI}}-{\mathrm{P}}_{\mathrm{E}\mathrm{S}-\mathrm{EE}}}{{\mathrm{V}}_{\mathrm{T}}} $$

Where P_ES-EI_ and P_ES-EE_ are respective P_ES_ determined at end-inspiratory and end-expiratory occlusion.

$$ {\mathrm{E}}_{\mathrm{L}}={\mathrm{E}}_{\mathrm{RS}}-{\mathrm{E}}_{\mathrm{CW}} $$

### Statistical analysis

Continuous variables are presented as the median and inter-quartile range. Data obtained in different experimental conditions were compared by the analysis of variance for repeated measure or Scheirer-Ray-Hare test as appropriate [28]. If significant, a Student’s *t* test or Mann-Whitney *U* test for paired data with Bonferroni correction for post-hoc multiple comparisons was applied for evaluating the differences between each experimental condition and the others. Spearman’s rank-order correlation was used to explore the relationship between ICP and CVP. Significance was established at *p* <  0.05. Analyses were performed with SPSS statistics software (V.23.0 IBM Corporation, New York, USA).

## Results

### Respiratory mechanics

There were significant differences in all respiratory mechanic parameters except for expiratory V_T_ among different experimental conditions at baseline PEEP level of 5 cm H_2_O (all *p* <  0.05, Table 1). Compared to the *Normal* condition, hydrochloride instillation significantly increased lung driving pressure and E_L_ in conditions with lung injury (*L*, *L + CW*, *L + CW + IH* and *L + IH* conditions); while P_ES-EE_, chest wall driving pressure and E_CW_ significantly increased in conditions with chest wall and abdomen strapping (*CW*, *L + CW* and *L + CW + IH* conditions, Table 1).

**Table 1:** Respiratory mechanics parameters at 5 cm H_2_O of positive end-expiratory pressure in each condition

	*Normal*	*CW*	*L*	*L + CW*	*L + CW + IH*	*L + IH*	*p* value
V_TE_ (ml)	134 (130.3, 173.8)	136 (131.3, 175)	136 (132.5, 177.5)	138 (131.8, 176)	136.5 (134.5, 175)	136.5 (133.5, 175.8)	> 0.999
P_PEAK_ (cm H_2_O)	18.5 (14.5, 21.5)	22 (17.5, 24.5)	23.5 (21.5, 24.8)*	30.5 (24.3, 32.8)*	27 (25.3, 35.8)*	22.5 (19.8, 25.5)	< 0.001
P_ES-EE_ (cm H_2_O)	4.1 (4.1, 5.1)	6.8 (5.4, 6.8)*	5.4 (4.1, 6.5)	7.5 (5.4, 10.2)*	6.8 (5.8, 9.2)*	5.4 (4.4, 5.4)	< 0.001
Airway resistance (cm H_2_O*s/L)	21.8 (17.5, 23.3)	21.2 (18, 27.8)	26.9 (20.6, 31.3)	34.9 (27.3, 39.5)*	30.4 (22, 42.9)*	23.2 (17.3, 30)	0.007
Airway driving pressure (cm H_2_O)	8.8 (6.8, 10.5)	12.2 (9.9, 13.6)*	13.6 (11.2, 13.6)*	19 (12.6, 20.1)*	15.6 (13.9, 22.8)*	12.9 (11.2, 13.6)*	< 0.001
Chest wall driving pressure (cm H_2_O)	2.7 (2.7, 4.1)	6.8 (4.4, 6.8)*	2 (1.4, 2.7)	4.8 (2.9, 5.4) *	6.1 (5.4, 6.8) *	2.7 (1.4, 2.7)	< 0.001
Transpulmonary driving pressure (cm H_2_O)	4.8 (4.1, 7.8)	5.4 (4.4, 7.8)	10.9 (9.5, 12.2)*	12.2 (9.7, 16.3)*	9.5 (8.5, 17)*	10.9 (8.5, 10.9)*	< 0.001
E_RS_ (cm H_2_O/L)	57.1 (48.1, 73.1)	79.3 (72.3, 96.4)	86.8 (77.6, 102.6)*	109.4 (95.4, 149.9)*	114.6 (84.7, 164.4)*	86.8 (78.4, 100)*	< 0.001
E_CW_ (cm H_2_O/L)	21.6 (20.3, 23.5)	38.9 (32.5, 49.6)*	13.1 (10.1, 18.6)*	28.1 (22.2, 39.6)*	40.3 (33, 48.1)*	17.6 (10.3, 20.4)	< 0.001
E_L_ (cm H_2_O/L)	37.5 (25.3, 50.2)	39 (31.3, 54.4)	74.2 (63.1, 89.1)*	84.4 (62.3, 112.9)*	70 (52.3, 119.5)*	65.6 (60.8, 85.7)*	0.001
E_CW_/E_RS_ ratio	0.4 (0.29, 0.48)	0.47 (0.41, 0.61)*	0.16 (0.1, 0.22)*	0.25 (0.21, 0.4)*	0.34 (0.29, 0.42)	0.21 (0.13, 0.27)*	< 0.001

Data are presented as median (interquartile range)

*p < 0.05 compared to the *Normal* condition

*V*_*TE*_ expiratory tidal volume, *P*_*PEAK*_ peak airway pressure, *P*_*ES-EE*_ end-expiratory esophageal pressure, *E*_*RS*_ respiratory system elastance, *E*_*CW*_ chest wall elastance, *E*_*L*_ lung elastance

**Fig. 2:**
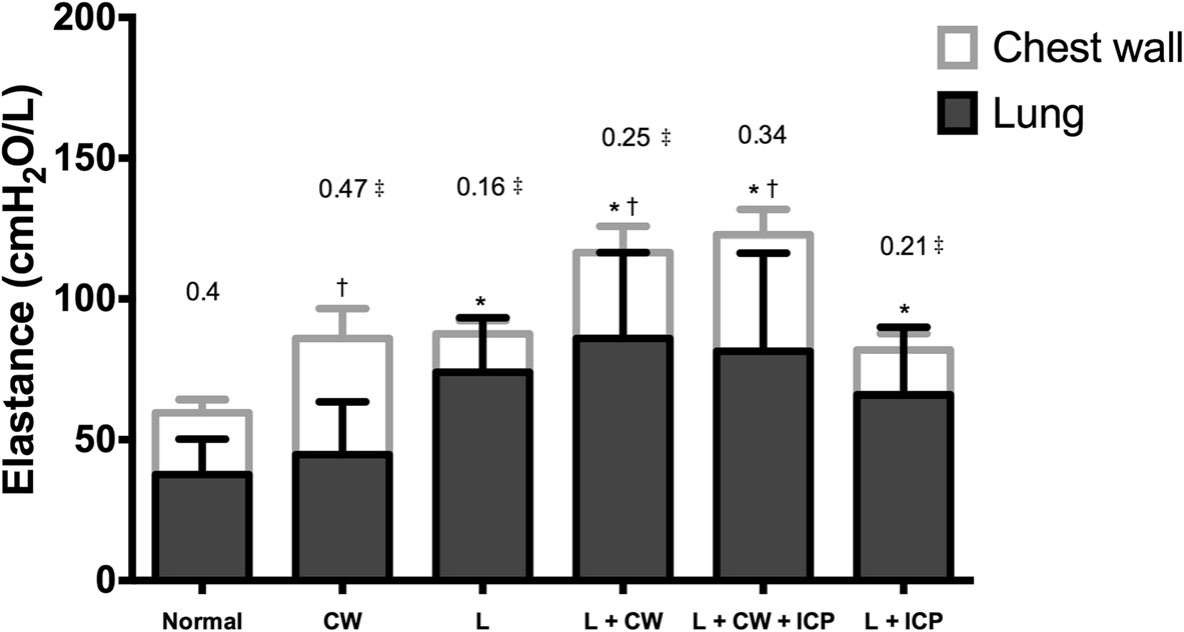
Stacked histograms of chest wall elastance and lung elastance. *: compared to the *Normal* condition, a significant greater lung elastance (E_L_) was observed in the conditions with lung injury. †: compared to the *Normal* condition, a significant greater chest wall elastance (E_CW_) was observed in the conditions with chest and abdomen strapping. The numbers on the top of each plot are the medians of the ratio of E_CW_ to respiratory system elastance. ‡: *p* < 0.05 compared to the *Normal* condition

The highest E_CW_/E_RS_ ratio was observed in the *CW* condition which was significantly higher than the *Normal* condition (*p* = 0.033, Table 1); while the lowest E_CW_/E_RS_ ratio was observed in the *L* condition which was significantly lower than the *Normal* condition (*p* = 0.035, Table 1). There was significant difference in E_CW_/E_RS_ ratio between the two conditions with IH (*p* = 0.004, Table 1). Figure 2 shows data of E_L_ and E_CW_, and the E_CW_/E_RS_ ratio.

### Changes of ICP in the non-IH conditions

**Fig. 3:**
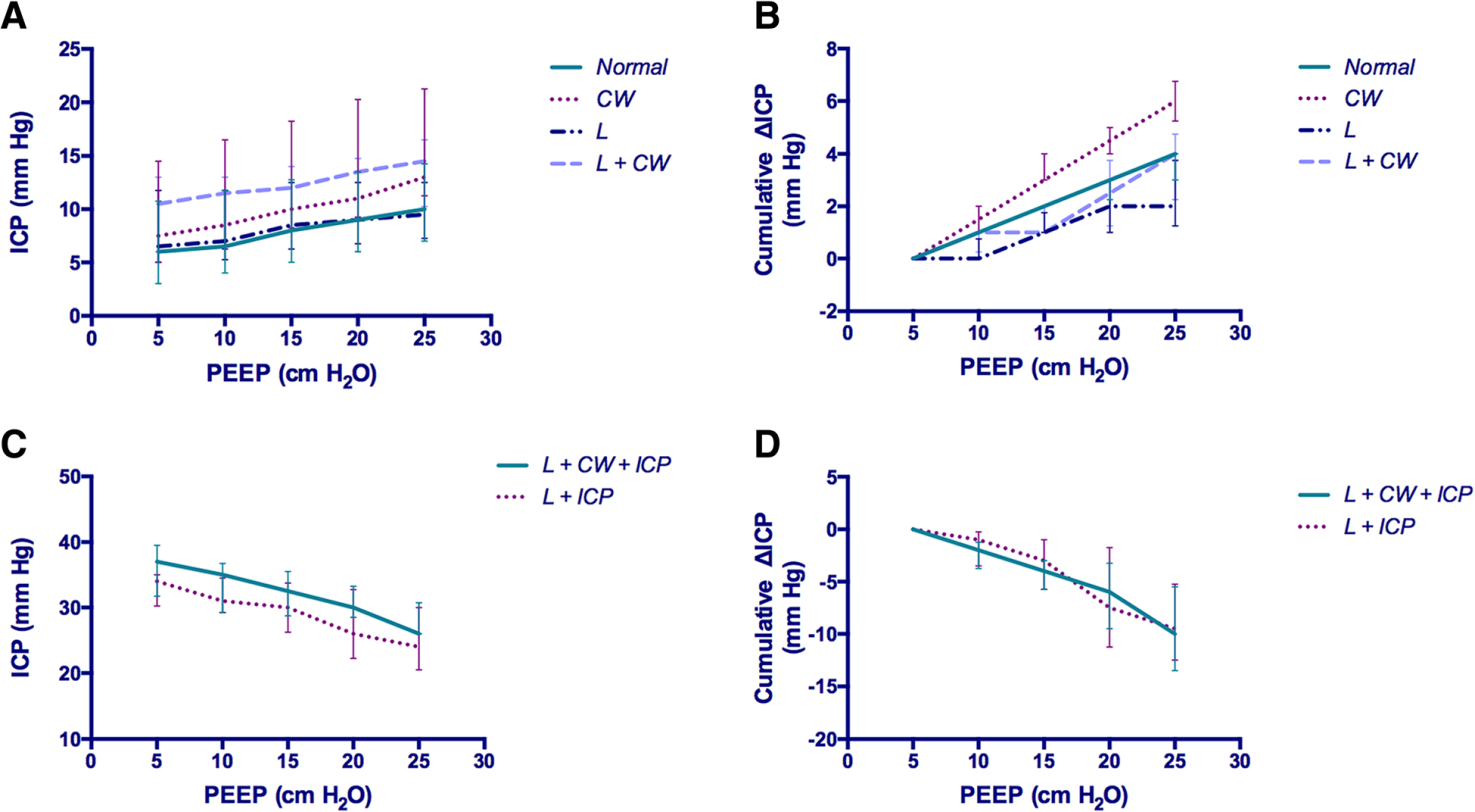
Changes of intracranial pressure with positive end-expiratory pressure increases. **a** Intracranial pressure (ICP) increased with positive end-expiratory pressure (PEEP) increases in all the conditions without intracranial hypertension (IH). **b** In the conditions without IH, the greatest cumulative change of ICP was observed in the *CW* condition, while the lowest one was observed in the *L* condition (both with statistical significance). **c** ICP decreased with PEEP increases in the conditions without IH. **d** No significant difference was observed in the change of ICP between the two conditions with IH

ICP significantly increased with increments of PEEP in all non-IH conditions (*p* <  0.001, Fig. 3a). The greatest cumulative increase in ICP was observed in the *CW* condition (6 [5.3, 6.8] mm Hg), which was significant higher than the other conditions (*p* value range: < 0.001 to 0.018). The lowest cumulative increase in ICP was observed in the *L* condition (2 [1.3, 3.8] mm Hg), which was significant lower than the other conditions (*p* <  0.001). There was no significant difference in cumulative ICP change between the *Normal* and the *L + CW* condition (4 [3.0, 4.0] versus 4 [2.3, 4.8] mm Hg, *p* >  0.999, Fig. 3b).

### Changes of ICP in the IH conditions

There was no difference in baseline ICP between the two IH conditions. ICP significantly decreased when PEEP was increased (*p* <  0.001, Fig. 3c). There was no significant difference in cumulative ICP change between the *L + CW + IH* and the *L + IH* conditions (− 10 [− 13.5, − 5.5] versus − 9.5 [− 12.5, − 5.3] mm Hg, *p* = 0.924, Fig. 3d). There was no significant difference in ICP change between each PEEP level (*p* = 0.389). Detailed ICP and the change of ICP at each PEEP levels was presented in Table 2.

**Table 2:** Intracranial pressure and the change of intracranial pressure at each positive end-expiratory pressure level

Condition	PEEP	ICP	∆ICP	Cumulative ∆ICP
	(cm H_2_O)	(mm Hg)	(mm Hg)	(mm Hg)
*Normal*	5	6 (3, 10.8)	–	–
	10	6.5 (4, 11.8)	1 (1, 1)	1 (1, 1)
	15	8 (5, 12.8)	1 (1, 1)	2 (2, 2)
	20	9 (6, 13.5)	1 (1, 1)	3 (2.3, 3)
	25	10 (7, 14.3)	1 (0.3, 1)	4 (3, 4)
*CW*	5	7.5 (5, 14.5)	–	–
	10	8.5 (6.3, 16.5)	1.5 (1, 2)	1.5 (1, 2)
	15	10 (8.3, 18.3)	2 (1, 2)	3 (3, 4)
	20	11 (9.3, 20.3)	1 (1, 1.8)	4.5 (4, 5)
	25	13 (11.3, 21.3)	1.5 (1, 2)	6 (5.3, 6.8)
*L*	5	6.5 (5, 11.8)	–	–
	10	7 (5.3, 11.8)	0 (0, 0.8)	0 (0, 0.8)
	15	8.5 (6.3, 12.5)	1 (1, 1)	1 (1, 1.8)
	20	9 (6.8, 12.5)	0.5 (0, 1.8)	2 (1, 3)
	25	9.5 (7.3, 12.5)	0 (0, 1)	2 (1.3, 3.8)
*L + CW*	5	10.5 (6, 13)	–	–
	10	11.5 (7, 19.8)	1 (0.3, 1)	1 (0.3, 1)
	15	12 (8, 14)	1 (0, 1)	1 (1, 2)
	20	13.5 (9.3, 14.8)	1 (0.3, 2)	2.5 (1.3, 3.8)
	25	14.5 (10.3, 16.5)	1 (1, 1)	4 (2.3, 4.8)
*L + CW + IH*	5	37 (31.8, 39.5)	–	–
	10	35 (29.3, 36.8)	-2 (−3.8, −1.3)	-2 (−3.8, −1.3)
	15	32.5 (28.8, 35.5)	-2 (−2, −1)	−4 (−5.8, − 3)
	20	30 (28.5, 33.3)	−1.5 (−3.5, 0)	−6 (−9.5, − 3.3)
	25	26 (26, 30.8)	−3.5 (−4, − 1.3)	−10 (− 13.5, − 5.5)
*L + IH*	5	34 (30.3, 35)	–	–
	10	31 (29.3, 34.5)	−1 (− 3.5, −0.3)	−1 (− 3.5, −0.3)
	15	30 (26.3, 33.8)	− 2 (− 2.8, − 0.3)	− 3 (− 5.8, − 1)
	20	26 (22.3, 32.8)	−2.5 (− 5.5, − 1)	−7.5 (− 11.3, − 1.8)
	25	24 (20.5, 30)	−2 (− 3.5, − 1.3)	−9.5 (− 12.5, − 5.3)

Data are presented as median (interquartile range)

*ICP* intracranial pressure, *PEEP* positive end-expiratory pressure

### Changes of hemodynamic parameters

**Fig. 4:**
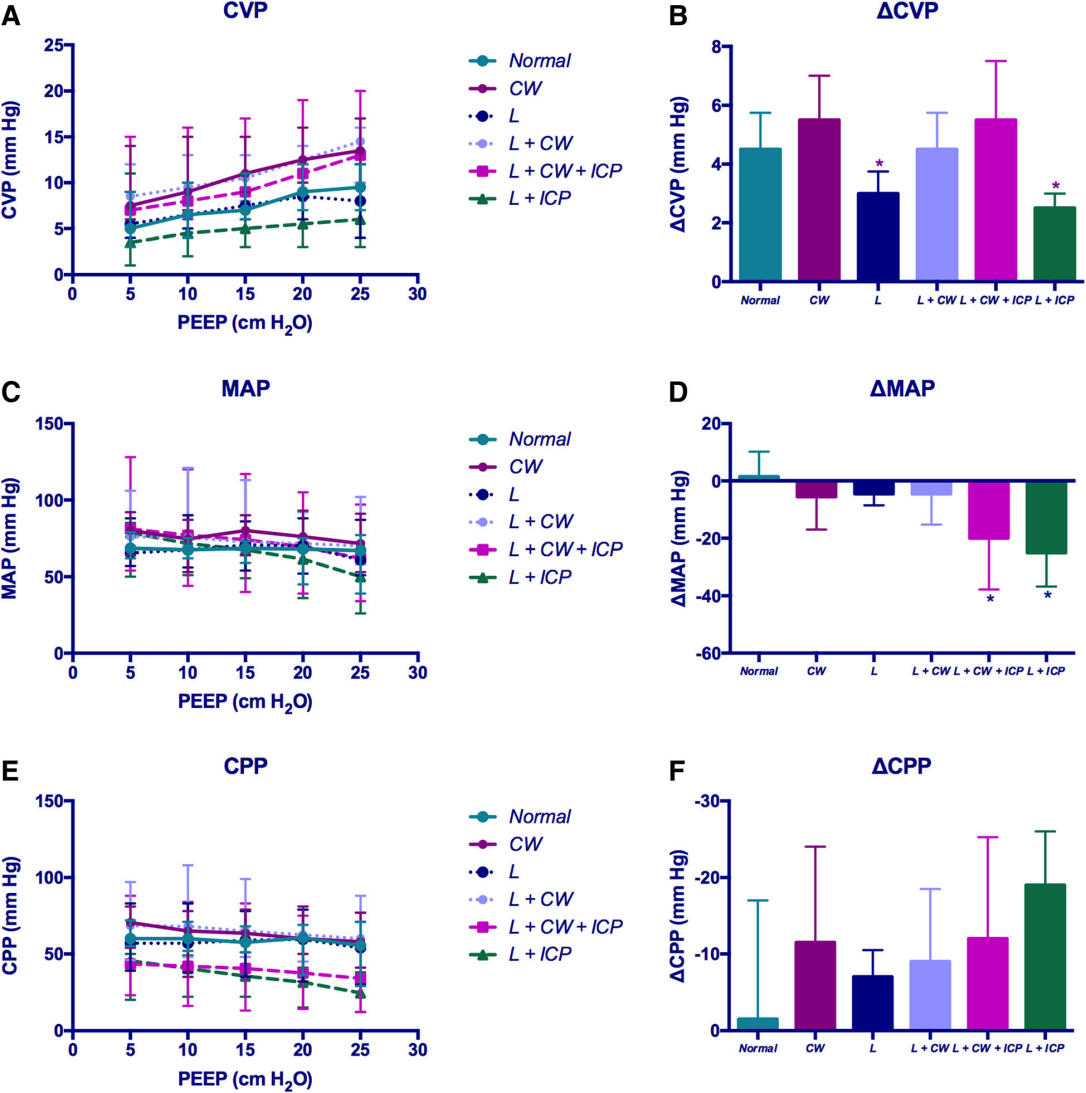
Change of hemodynamic parameters with positive end-expiratory pressure increases. **a** Central venous pressure (CVP) increased with positive end-expiratory pressure (PEEP) increases in all the conditions. CVP was significantly higher in the conditions with chest wall and abdomen strapping. **b** The change of CVP was significantly lower in the conditions with only lung injury (*L* and *L + IH* conditions). **c** Mean arterial pressure (MAP) decreased with PEEP increases in all the conditions. **d** The change of MAP was significantly greater in the conditions with intracranial hypertension (IH). **e** Cerebral perfusion pressure (CPP) decreased with PEEP increases in all the conditions. CPP was significantly lower in the conditions with IH. **f** No significant difference was observed in the change of CPP between the conditions

Baseline CVP values were significantly higher in the conditions with chest wall strapping (*CW*, *L + CW* and *L + CW + IH* conditions) than those without chest wall strapping (*Normal, L* and *L + IH* conditions*, p* value range < 0.001 to 0.038, Fig. 4a). CVP significantly increased as increments of PEEP in each condition, with a significant different extent of CVP increase (*p* < 0.001); the increases of CVP in the *L* and the *L + IH* conditions were significantly lower than those in other conditions (*p* value range 0.001 to 0.020, Fig. 4b). ICP was significantly correlated to CVP in the non-IH conditions (*r* = 0.654, *p* < 0.001); however, ICP and CVP were no longer correlated in the IH conditions (*r* = − 0.066, *p* = 0.561). There was no significant difference in baseline MAP among different conditions (*p* = 0.125, Fig. 4c). MAP significantly decreased when PEEP was increased in each condition, whereas the decrease of MAP in the IH conditions were significantly greater than those in other conditions (*p* value range 0.007 to 0.025); but no significant difference was observed between the two IH conditions (*p* = 0.961, Fig. 4d). CPP values in the IH conditions were significantly lower than those in other conditions (*p* value range < 0.001 to 0.010, Fig. 4e). There was no significant difference in the change of CPP among different conditions (*p* = 0.642, Fig. 4f).

## Discussion

The main findings of this preliminary study were:

1) E_L_ can be increased by hydrochloride induced lung injury, accompanied with a decreased E_CW_/E_RS_ ratio. In the contrast, E_CW_ can be increased by chest wall and abdominal strapping, accompanied by increased E_CW_/E_RS_ ratio. 2) In pigs without IH, ICP increases as increasing of PEEP. The extent of ICP increase was determined by respiratory mechanics. The effect of PEEP on ICP was enhanced in increased E_CW_ and E_CW_/E_RS_ ratio condition, whereas the effect of PEEP on ICP was attenuated in increased E_L_ and thereby reduced E_CW_/E_RS_ ratio condition. 3) In pigs with IH, however, the increase of PEEP reduced ICP. The difference of respiratory mechanics has nothing to do with the effect of PEEP on ICP under IH conditions.

The Monro-Kellie doctrine suggests that with an intact skull, the combined volume of the brain, the blood and the cerebrospinal fluid is constant and determines the ICP [29]. An increase in volume of single component causes a decrease in volume of remaining one or both of the two components, in a certain degree, to keep ICP remained in a normal range. ICP will increase rapidly once compensation is exhausted. The PEEP-induced increase of pleural pressure may be transmitted to the intracranial cavity directly or may reduce cerebral venous drainage and eventually increase ICP [18, 30], where the pleural pressure serves as an intermediate link from the lung to the cranium.

The transmission of PEEP into the pleural cavity dependents on the respiratory mechanics of the lung and the chest wall. P_AW_ equals the sum of transpulmonary pressure and pleural pressure when the airway resistance is nil (i.e. no airflow in the airway). In this situation, the distribution of P_AW_ to pleural cavity depends on the E_CW_/E_RS_ ratio [21]. In other words, a higher E_CW_/E_RS_ ratio could lead to a greater impact of PEEP on pleural pressure, which might in turns result in a greater increase of ICP. This was supported by our data: in animals without IH, the effect of PEEP on ICP became obvious under condition of increased E_CW_/E_RS_ ratio, but was attenuated under condition of decreased E_CW_/E_RS_ ratio (Fig. 3a and b).

Surprisingly, however, our data suggested that the increase of PEEP reduces ICP in animals with IH. This might be explained by the change of cerebral blood volume. In our study, an intracranial balloon was inflated to induce IH mimicking that caused by space occupying lesions. The compensatory potential of cerebrospinal fluid was exhausted and thereby the volume in the skull was predominantly determined by the intracranial blood volume in this situation since the brain is almost incompressible and has a relatively constant volume.

An increased cerebral blood volume can be caused by reduced cerebral venous drainage and/or increased arterial perfusion. The application of PEEP can reduce cerebral venous drainage as we discussed above, results in increase of cerebral venous blood volume and elevation of ICP. However, this effect might become less obvious in the IH conditions. In experimental studies conducted in dogs, it was found that the increase of ICP due to PEEP was diminished in the presence of IH, which can be explained by the Starling resistor or waterfall concept [31, 32]. McGuire et al. found a similar phenomenon in a clinical study [11], in which ICP increased in patients with normal baseline ICP but did not significantly change in patients with elevated ICP when a maximal PEEP of 15 cm H_2_O was applied. We speculated that the effect of PEEP on ICP from the venous side in IH pigs in the present study might be also diminished. This is supported by our data: ICP was correlated to CVP, which represents the downstream (venous returning) impedance, in the non-IH conditions; however, no such correlationshiop was observed in the IH conditions, which means that the change of ICP was more likely determined by the change of cerebral perfusion in the IH conditions.

The normal brain has several mechanisms for regulating cerebral blood flow and volume, referred as cerebral autoregulation. Under physiological conditions, vessels in the brain can regulate the vascular tone to maintain a constant cerebral blood flow in MAP between 60 and 160 mmHg [33, 34]. However, recent studies suggest an asymmetric dynamic cerebral autoregulatory response that the autoregulatory ability appears to be more effective in buffering increases in MAP and CPP as compared to reductions [35–37]. In the IH conditions in this experiment, MAP and CPP decreased with PEEP increases. In addition, when high PEEP levels were applied, CPP dropped to a low range that was beyond the capacity of autoregulation mechanisms to maintain a constant cerebral blood flow. Therefore, it is reasonable to infer that the cerebral blood flow decreased when PEEP was increased, although we did not measure the actual cerebral blood flow in the present study. The decrease of cerebral blood flow resulted in decrease of cerebral blood volume and eventually resulted in decrease of ICP. Since there was no significant difference in MAP and CPP between the two conditions of IH (although with different respiratory mechanics), no difference was found in the change of ICP.

In the present experiment we provided sedation rather than anesthesia by midazolam and fentanyl infusion. Unlike general anesthesia (inhaled anesthetics or i.v. barbiturates), which is usually considered as a salvage therapy for refractory intracranial hypertension, sedation is more likely to be chosen in the clinical practice (in the ICUs) for intracranial hypertensive patients. Therefore, we used midazolam and fentanyl infusion (to provide sedation) instead of general anesthesia.

### Limitations

Due to the nature of a preliminary study, there were many limitations in this study. First, we did not measure/control PaCO_2_ in this study. Therefore, the influence of PaCO_2_ on ICP cannot be excluded. Second, animals were not randomized to a certain condition. Instead, we tested all the conditions in sequence in each animal. Third, we used CPP as the indicator of cerebral perfusion. Although strong correlated, CPP does not always reflect cerebral perfusion. Measurement of cerebral blood flow is needed for full understanding of the effects of PEEP in intracranial hemodynamics in future studies.

## Conclusions

Different respiratory mechanics models can be established via hydrochloride induced lung injury and chest wall and abdominal strapping. For pigs with normal ICP, the effect of PEEP on ICP becomes more obvious when the E_CW_/E_RS_ ratio increases, and is attenuated when the ratio decreases. For pigs with IH, the responsiveness of ICP to PEEP is independent of respiratory mechanics and likely depends to a greater extent on the effect of PEEP on hemodynamics.

## Abbreviations

ARDS: Acute respiratory distress syndrome
CPP: Cerebral perfusion pressure
CVP: Central venous pressure
E_CW_: Chest wall elastance
E_L_: Lung elastance
E_RS_: Respiratory system elastance
ICP: Intracranial pressure
IH: Intracranial hypertension
MAP: Mean arterial pressure
P_AW_: Airway pressure
PEEP: Positive end-expiratory pressure
P_ES_: Esophageal pressure
P_ES-EE_: End-expiratory esophageal pressure
P_ES-EI_: End-inspiratory esophageal pressure
P_PEAK_: Peak airway pressure
V_TE_: Expiratory tidal volume
